# Cyanoacetamidobetaine—A Zwitterionic Nitrile Derivative

**DOI:** 10.1002/open.202500485

**Published:** 2025-10-09

**Authors:** Pan Duan, Julia‐Maria Hübner, Florian Puls, Vitaliy Romaka, Hans‐Joachim Knölker, Michael Ruck

**Affiliations:** ^1^ Faculty of Chemistry and Food Chemistry TUD Dresden University of Technology 01062 Dresden Germany; ^2^ Sächsische Akademie der Wissenschaften zu Leipzig Karl‐Tauchnitz‐Straße 1 04107 Leipzig Germany; ^3^ Max Planck Institute for Chemical Physics of Solids Nöthnitzer Straße 40 01187 Dresden Germany

**Keywords:** betaine derivate, crystal structure analysis, density functional theory, zwitterions

## Abstract

Cyanoacetamidobetaine, or *N*‐cyano‐2‐(trimethylammonio)acetaminide, is obtained from a reaction of sodium dicyanamide and betainium chloride in an aqueous solution after 4 h at 30 °C. By adding DCM, single‐crystals suitable for diffraction experiments are obtained. The compound crystallizes in the monoclinic space group *P*2_1_/*c* with lattice parameters *a* = 7.3365(2) Å, *b* = 8.8351(3) Å, *c* = 11.0977(4) Å, and *β* = 91.948(2)°. The unexpected reaction product comprises a trimethylammonium head group linked via a methylene bridge to a negative cyanoacetaminide moiety, highlighting the thermodynamic preference for intramolecular charge compensation over salt formation of betainium with dicyanamide. The findings are supported by infrared and nuclear magnetic resonance spectra and density functional theory calculations.

## Graphical abstract

The reaction of sodium dicyanamide with betainium chloride in aqueous solution affords *N*‐cyano‐2‐(trimethylammonio)acetaminide (cyanoacetamidobetaine). Single crystal X‐ray diffraction reveals a monoclinic structure comprising a trimethylammonium head group connected to a cyanoacetaminide unit via a methylene bridge. The structure and bonding are corroborated by infrared and nuclear magnetic resonance spectroscopy as well as density functional theory calculations.

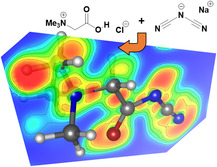

## 1 Introduction

Ionic liquids (ILs), which are by definition salts with a melting point below 100 °C, have attracted attention as their negligible vapor pressure, broad electrochemical windows, and tailorable polarity make them appealing alternatives to conventional solvents. Importantly, the wide range of cation–anion combinations allow not only for the fine‐tuning of properties, but lead to the development of task‐specific ILs with the ability to dissolve a variety of metal oxides with tremendous significance both for primary metal production and recycling.^[^ ^1^, ^2^, ^3^, ^4^, ^5^, ^6^, ^7^ ^–^ ^8^ ^]^ In particular, [Hbet][NTf_2_] (betainium bis(trifluoromethylsulfonyl)imide) has been shown to dissolve a wide range of metal oxides—including oxides of zinc, copper, rare earths, and lead at elevated temperatures (≈175 °C) in water‐free systems, forming stable betaine‐metal complexes.^[^ ^9^, ^10^ ^–^ ^11^ ^]^ Its performance is largely due to the coordination capability of the carboxylic group in the [Hbet]^+^ cation, which can interact directly with metal centers. Moreover, betaine is a natural, inexpensive, and biodegradable compound derived from sugar beet processing, aligning well with the principles of green chemistry. However, despite these advantages, [Hbet][NTf_2_] suffers from drawbacks linked to the [NTf_2_]^−^ anion, which is fluorinated, persistent, and associated with toxicity and environmental concerns. Therefore, identifying fluorine‐free, eco‐friendly anions that preserve the metal‐dissolving capabilities of [Hbet][NTf_2_] is of considerable interest.

Dicyanamide (DCA^−^) is one such candidate. Several ILs with conventional cations (e.g., imidazolium, pyrrolidinium, phosphonium) exist,^[^ ^12^, ^13^ ^–^ ^14^ ^]^ exhibiting improved viscosity compared to ILs comprising BF_4_^–^, Cl^–^, PF_6_^–^ as anions,^[^ ^15^, ^16^ ^–^ ^17^ ^]^ meanwhile showing enhanced electrochemical windows^[^ ^18^ ^]^ and extraction abilities.^[^ ^15^ ^,^ ^19^, ^20^, ^21^ ^–^ ^22^ ^]^ As these cations are in general rather expensive and show a limited biodegradability, a betaine‐based dicyanamide IL could represent a promising alternative, aiming to combine metal oxide dissolution capacity with a more benign anion.^[^ ^23^, ^24^ ^–^ ^25^ ^]^

With this in mind, we set out to develop a new [Hbet]‐derived IL by replacing [NTf_2_]^−^ with DCA^−^. Rather than obtaining a new IL, our synthesis unexpectedly yielded a crystalline zwitterionic ammonium acetaminide compound, whose crystal structure, spectroscopic properties and possible formation mechanism are presented herein.

## 2 Results & Discussion

**Scheme 1:**
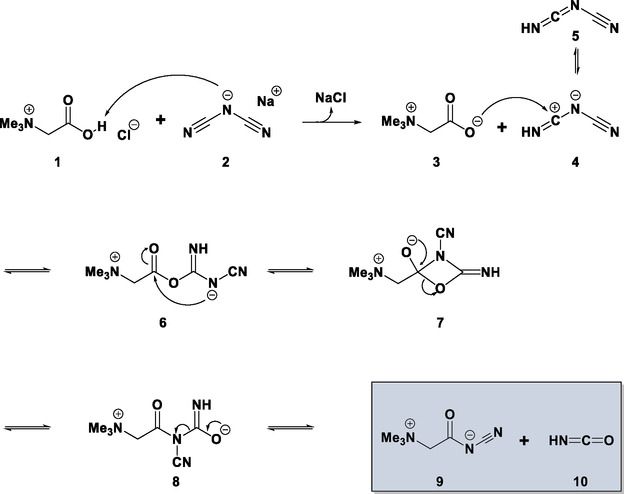
Reaction pathway proposed for the formation of cyanoacetamidobetaine.

The compound cyanoacetamidobetaine was formed from an aqueous solution of betainium chloride and sodium dicyanamide. A potential reaction pathway is shown in **Scheme** **1**.

The first step is the protonation of the dicyanamide anion **2** at the nitrile nitrogen,^[^ ^26^, ^27^ ^–^ ^28^ ^]^ forming a dicyanamine **5**. The proton reversibly transfers to the terminal cyano group to form an electrophilic carbon atom in **4**. Subsequently, the oxygen atom of the carboxylate in molecule **3** attacks the electrophilic carbon.^[^ ^26^ ^]^ The nitrogen atom then attacks the carbonyl carbon atom to form a 1,3‐oxazetidine. Ring opening proceeds by cleavage of the carbon–oxygen bond. Finally, the target product and the by‐product isocyanic acid are generated, as similarly observed in other reactions.^[^ ^29^ ^,^ ^30^ ^]^

**Figure 1:**
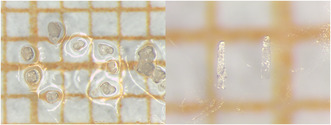
Cyanoacetamidobetaine before (left) and after (right) adding DCM. Square sizes correspond to 1 mm divisions.

The by‐product NaCl was removed by washing with ethanol. After solvent removal, an oil‐like substance remains, in which transparent crystal agglomerates form after a few hours of storage at room temperature (**Figure** **1**, left). By addition and subsequent removal of DCM, transparent single crystals with a lath‐like habit are obtained (Figure 1, right).

Single‐crystal X‐ray diffraction revealed that, although several specimens were screened, all candidates deemed suitable by optical microscopy assessment were either twinned or composed of multiple crystallites. Attempts to cleave larger specimens resulted in significant degradation of crystal quality. Therefore, structure determination was carried out on a twinned crystal (Figure S1, Supporting Information), with the second twin component contributing 26.8% to the total intensity. The compound crystallizes in the monoclinic space group *P*2_1_/*c* with lattice parameters *a* = 7.3365(2) Å, *b* = 8.8351(3) Å, *c* = 11.0977(4) Å, and *β* = 91.948(2)°. The crystal exhibited twinning, with the twin operation corresponding to a 180° rotation around the *a*‐axis (twin matrix: 100|0–10|00–1). This operation is not a symmetry element of the monoclinic Laue class, and the small deviation of *β* from orthogonality points to the origin of the nonmerohedral twinning. The structure refinement resulted in residuals *R*_1_ = 0.039, *wR*_2_ = 0.097 (for further information on data collection and refinement details, see Table S1–S3, Supporting Information).

All reflections of the powder X‐ray diffractogram (Figure S2, Supporting Information) can be indexed on the basis of the structure obtained from single‐crystal diffraction. The lattice parameters obtained by a Le Bail fit to the powder diffraction data measured at room temperature [*a* = 7.3375(3) Å, *b* = 9.0505(4) Å, *c* = 11.2709(5) Å, and *β* = 91.778(2)°] are in reasonable agreement with those of the single‐crystal at 100 K.

**Figure 2:**
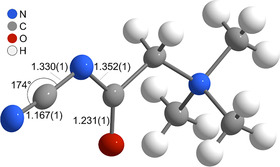
Asymmetric unit of the crystal structure of cyanoacetamidobetaine with selected interatomic distances in  Å and bond angles. The ellipsoids, shown for nonhydrogen atoms, enclose a space in which the electron density of the atoms at 100 K can be found with 75% probability.

The crystal structure reveals a zwitterionic molecule in the asymmetric unit, comprising a trimethylammonium head group connected through a methylene bridge to a cyanoacetaminide moiety (**Figure** **2**). All N^+^–C distances amount to ≈1.50 Å as similarly observed for other compounds featuring trimethylammonium groups.^[^ ^31^ ^]^ The carbonyl C=O bond length is 1.23 Å, consistent with a typical amide carbonyl (ca. 1.22–1.24 Å).^[^ ^32^ ^]^ The adjacent C—N^−^ bond, connecting the deprotonated amide N atom to the central carbon atom, measures 1.33 Å, which is slightly shorter than a standard C—N single bond (ca. 1.36–1.38 Å), suggesting partial double‐bond character due to resonance delocalization. The N^−^—C bond connecting the anionic nitrogen atom to the cyano‐bearing carbon atom is also 1.33 Å long. Therefore, it is similar in length and likewise indicative of conjugation. The C≡N triple bond measures 1.16 Å, well within the expected range for a nitrile group (1.15 to 1.17 Å),^[^ ^32^ ^]^ confirming its unperturbed nature (for interatomic distances, see Table S4, Supporting Information). Overall, the bond metrics support a delocalized electronic structure over the amidonitrile fragment, stabilizing the anionic resonance form.

The bond angles (Figure 2 and Table S4, Supporting Information) around the quaternary ammonium nitrogen atom (N^+^) range from 107°–112°, consistent with near‐tetrahedral geometry (regular angle 109.5°). These values are similar to those found in trimethylammonium halide structures as in trimethylammonium bromide with C—N—C angles reported between 108° and 110°.^[^ ^33^ ^]^ Moving along the molecular backbone, the C—C—N (116°) and N—C—C (110°) angles reflect partial sp^2^ character at the carbonyl‐bearing carbon atom, in line with angle expansions in cyanoacetamide derivatives.^[^ ^34^ ^]^ Importantly, the C—N—C (amidic) angle of 116° and the nearly linear N—C—N angle of 174° match well with the 172°–174° angles observed in alkali metal dicyanamide salts (e.g., NH_4_[N(CN)_4_]),^[^ ^35^ ^]^ highlighting strong conjugation and resonance delocalization across the NCN unit.

**Figure 3:**
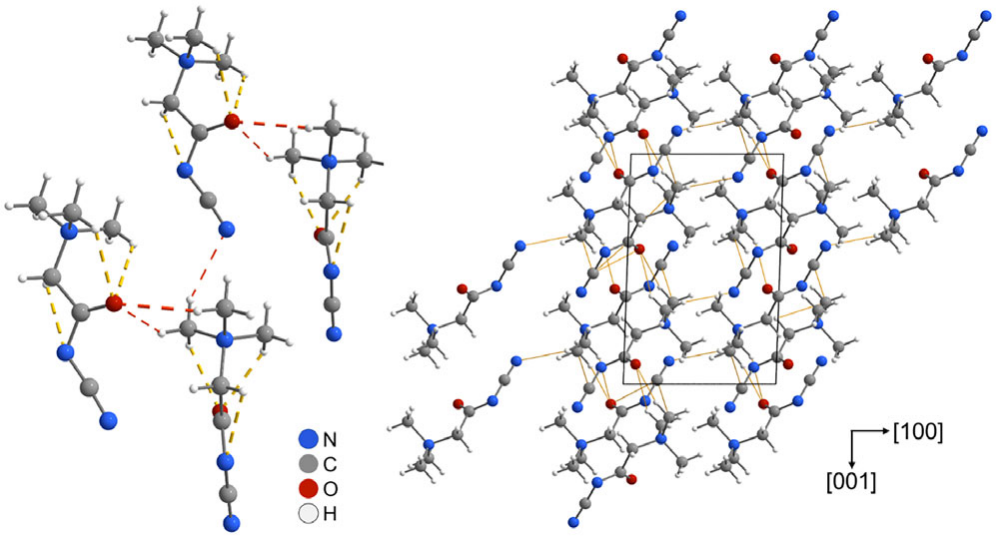
Molecular packing in the crystal structure of cyanoacetamidobetaine. Left: intramolecular H···*A* (*A* = O, N) bonds up to 2.40 Å shown in yellow, intermolecular ones up to 2.60 Å in red. View along [111]. Right: arrangement within unit cell with weak H···*A* bonds shown in yellow, view along [010].

The molecular entities exhibit several weak hydrogen bonds of the type H···*A* (*A* = O, N), both intra‐ and intermolecularly, contributing to the overall packing arrangement (**Figure** **3**, right). Intramolecular interactions include two H···O contacts of 2.35 and 2.40 Å, and one H···N^−^ contact of 2.48 Å (yellow dashed lines in Figure 3, left). These distances are typical for C—H···O or C—H···N interactions in betaine derivatives, which often range from 2.30 to 2.60  Å in similar systems.^[^ ^36^, ^37^ ^–^ ^38^ ^]^ Intermolecular hydrogen bonds (red dashed lines in Figure 3, left) are slightly longer, with H···O distances of 2.50 and 2.56 Å, and an H···N contact of 2.57 Å. This increase is consistent with weak, packing‐stabilizing hydrogen bonds due to reduced preorganization and greater geometric flexibility in intermolecular settings.

The formation of cyanoacetamidobetaine as a zwitterion, rather than an IL composed of [Hbet]^+^ and [DCA]^−^, highlights the thermodynamic preference for intramolecular charge compensation in this system. Zwitterionic compounds featuring betaine‐type structures with strongly electron‐withdrawing groups, such as cyano and carbonyl functionalities, are known to exhibit enhanced stability due to resonance delocalization and favorable hydrogen bonding. Related systems, including pyridinium‐derived zwitterions and cyanopropenide‐based structures, have similarly been shown to favor zwitterionic over ionic forms in the solid state, particularly in case the counter anion lacks sufficient basicity or internal stabilization dominates.^[^ ^39^, ^40^, ^41^, ^42^, ^43^, ^44^, ^45^ ^–^ ^46^ ^]^

**Figure 4:**
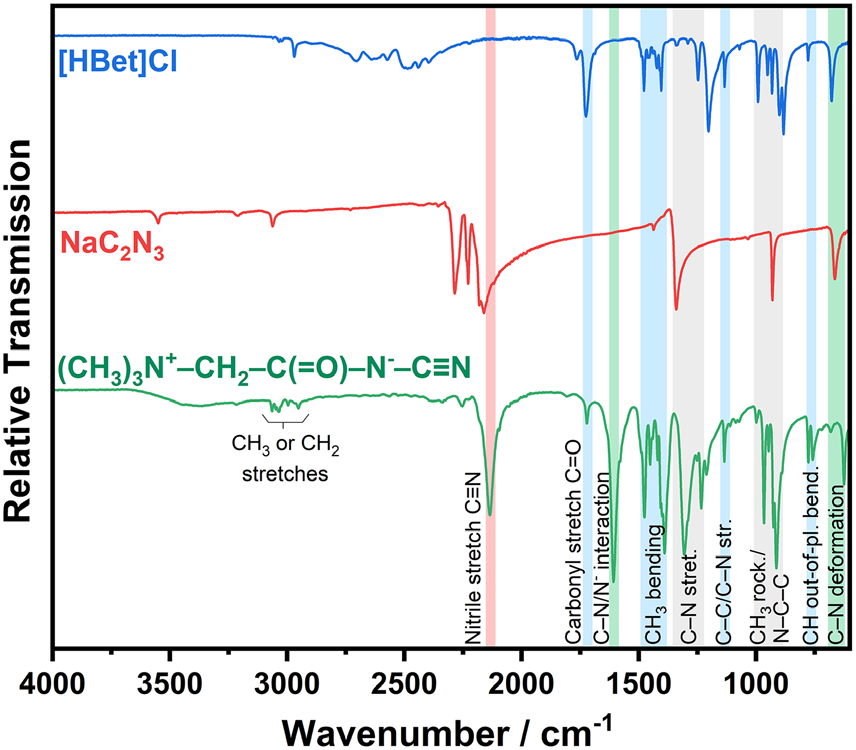
IR spectra of cyanoacetamidobetaine (green), sodium dicyanamide (red), and HbetCl (blue). Bands found in the parent compounds are denoted in red and blue, respectively.

The infrared (IR) spectrum of the compound supports the structural assignment derived from single‐crystal X‐ray diffraction. A strong absorption band at 2133 cm^−1^ corresponds to the stretching vibration of the nitrile (C≡N) group, in agreement with the characteristic range for the dicyanamide anion (N(CN)_2_^−^) as observed experimentally (**Figure** **4** and Table S5, Supporting Information) and reported for its potassium and rubidium salts (2130 to 2140 cm^−1^).^[^ ^47^ ^]^ The carbonyl (C=O) stretching vibration appears at 1722 cm^−1^ similar as in betainium chloride (HbetCl)^[^ ^48^ ^]^ and characteristic of an amide carbonyl. The band at 1606 cm^−1^ can be assigned to N—C bending, in line with the amide moiety observed crystallographically. Importantly, the IR data support the existence of a zwitterionic structure, as inferred from the crystal structure: the lack of broad O—H or N—H^+^ stretches and the presence of distinct C=O and C≡N bands point toward a neutral amide with an internal negative charge on nitrogen, rather than a protonated amine or carboxylate. This interpretation is further confirmed by the bond lengths observed in the crystal structure, particularly by the short C—N bonds (≈1.33 Å) and the planarity of the amidonitrile fragment, consistent with resonance delocalization across the —C(=O)—N^−^—C≡N *π*‐system. The experimentally obtained IR spectrum is in good agreement with the one calculated based on a density functional theory (DFT) optimized structure (Figure S5, Supporting Information).

Solid state nuclear magnetic resonance (NMR) ^1^H MAS NMR (Figure S3, Supporting Information) denotes CH_3_, and CH_2_, and ^13^C CP/MAS NMR (Figure S4, Supporting Information) indicates CH_3_, CH_2_, C≡N, and C=O environments and is, therefore, fully consistent with the results from single‐crystal structure analysis (Figure 2) and IR spectroscopy (Figure 4).

The atomic structure was fully relaxed using DFT, and the resulting coordinates show good agreement with the experimental crystal structure (Table S6, Supporting Information).

**Figure 5:**
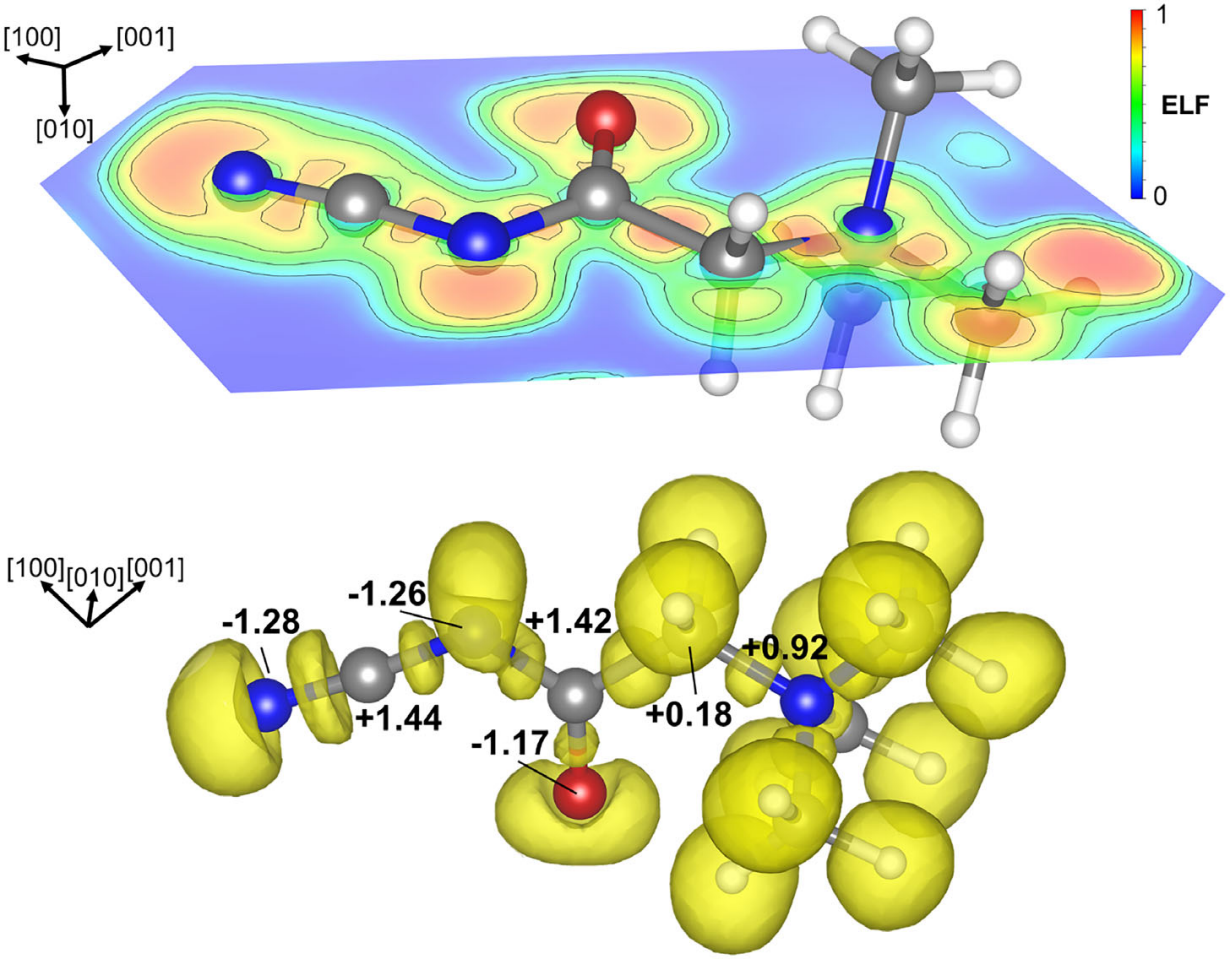
Visualization of the ELF for the optimized molecular structure (top) and the ELF isosurface at 0.78 (below).

Electron localization function (ELF) analysis was carried out to examine the spatial distribution of localized electron density, providing insights into both bonding and lone‐pair interactions. The ELF color map projected onto the molecular surface shows values approaching 1 near the terminal nitrogen atom, the nitrogen situated between two carbon atoms, and the oxygen atom (Figure 5, top), consistent with regions of strong electron localization. Complementarily, the ELF isosurface at a value of 0.78 (**Figure** **5**, bottom) reveals valence basins between bonded atoms, corresponding to covalent bonds. In addition, localized ELF maxima near the same nitrogen and oxygen atoms indicate the presence of nonbonding valence basins, associated with lone electron pairs on these electronegative atoms.

Bader charge analysis (Figure 5, bottom, Table S7, Supporting Information) reveals a clear correlation between atomic charges and the local chemical environment in the optimized molecular structure. Nitrogen atoms display a wide range of electron populations depending on their bonding context: a quaternary nitrogen atom bonded to four carbon atoms carries a net positive charge of +0.92, consistent with electron donation and partial oxidation. In contrast, nitrogen atoms bonded to one or two carbon atoms show net charges of −1.28 and −1.26, respectively, indicating significant electron accumulation. However, despite a triple bond between the C atom and the end standing N atom could be expected based on the interatomic distances (N3–C1: 1.16 Å), the net charges of both N atoms of the NCN group are similar, indicating delocalized electrons in a *π*‐system. O atoms exhibit a net charge of −1.17, consistent with their strong electronegativity and electron‐withdrawing character.

The C atoms also show considerable charge variation. The ones bonded to multiple electronegative atoms are strongly electron‐deficient, with net charges of +1.44 (two N neighbors) and +1.42 (bonded to N, O), reflecting their central roles in polar covalent bonding environments. In contrast, the C atoms in the methyl groups (+0.11 to +0.14) and the methylene group (+0.18) have only small positive charges. Overall, the Bader charge distribution supports a chemically intuitive picture of bonding and electron distribution across the molecule.

Bonding analysis based on ICOBI and –ICOHP values obtained from LOBSTER (Table S8, Supporting Information) confirms the presence of strong multiple bonds, such as a N≡C triple bond (ICOBI = 2.51, –ICOHP = 19.72 eV) and a C=O double bond (ICOBI = 1.57, –ICOHP = 16.29 eV), alongside typical single bonds in aliphatic environments (ICOBI ≈ 0.93, –ICOHP ≈ 9 eV). These findings are consistent with the electronic structure and geometry revealed by ELF and Bader charge analysis.

For comparison, the structure of the hypothetical compound (CH_3_)_3_—N^+^—CH_2_—(C=O)—O—C≡N was optimized as well. However, as the DFT structure optimization resulted in the decomposition of the molecule, it was concluded that this compound is not obtained as intermediate. Therefore, reaction pathways including this hypothetical compound were ruled out.

As the compound contains an anionic amidine‐nitrile, it poses a risk due to the chemical activity of the anionic N^−^ as well as the possibility of cyanide release upon metabolization, and the toxicity was, therefore, predicted as high‐risk (class III) by the Toxtree software v3.1.0 based on the Cramer rule and structural alerts.^[^ ^49^ ^]^ To explore the risk in more detail, the absorption, distribution, metabolism, excretion, and toxicity, the so‐called ADMET‐properties were predicted using the online‐tool ProTox 3.0^[^ ^50^ ^]^ and the program VEGA QSAR.^[^ ^51^ ^]^ The prediction hints toward activities in terms of ecotoxicity and neurotoxicity with the potential of the compound passing the blood–brain barrier. Therefore, as the compound poses the risk of toxicity, no further experiments were conducted.

In this work, a novel cyanoacetamidobetaine compound was synthesized. The zwitterionic molecule features a methylene bridge to a cyanoacetamide moiety. To the best of our knowledge, the quaternary ammonium cyanoacetamidobetaine scaffold—and potential derivatives such as ester, cyclic urea, or thioamide variants—are novel and would, therefore, pose a versatile building block platform for ionic derivatives offering, due to the zwitterionic nature, water solubility and bioavailability‐modifying potential.

## 3 Experimental Section

3.1

3.1.1

##### Synthesis

Sodium dicyanamide (NaDCA, 96%) was obtained from Merck. Betainium chloride ([Hbet]Cl, 99%) was purchased from abcr GmbH. NaDCA (93.5 mg, 1.05 mmol) was added to a 1 m aqueous solution of [Hbet]Cl (153.6 mg, 1.00 mmol). The mixture was heated for 4 h at 30 °C under constant stirring in air. Subsequently, water was removed in a rotary evaporator at 50 °C. Then, ethanol was added and the mixture was filtered to remove the solid by‐product NaCl. The residual ethanol contained in the filtrate was subsequently removed by rotary evaporation at 50 °C, resulting first in a transparent oil‐like phase. Transparent crystals form after storage at room temperature overnight. To obtain high‐quality crystals suitable for single crystal X‐ray diffraction, DCM was added and subsequently removed by rotary evaporation at 50 °C. Again, after storage at room temperature overnight, the product crystallized.

##### Single Crystal X‐Ray Diffraction

Data was measured on a Rigaku Synergy S diffractometer with Mo‐Kα radiation (*λ* = 0.71073 Å). The device was equipped with a hybrid pixel array detector (Dectris Eiger2 1 M) and measurements were carried out at 100 K. Data integration and scaling were performed using CrysAlisPro v.42.49.^[^ ^52^ ^]^ The monoclinic crystal was identified as twinned, and the appropriate twin law was applied during data reduction. A multidomain integration was carried out, and absorption correction was applied using the scale3 abspack algorithm. The resulting HKLF 5 file, containing merged reflections with twin domain information, was used for structure solution and refinement in JANA2020.^[^ ^53^ ^]^ The structure was solved using Superflip,^[^ ^54^ ^]^ and twin fractions were refined as part of the structural model using the twin refinement procedure in JANA2020. Deposition umber 2,478,860 contains the supplementary crystallographic data for this paper. These data are provided free of charge by the joint Cambridge Crystallographic Data Centre and Fachinformationszentrum Karlsruhe Access Structures service.

##### Powder X‐Ray Diffraction (PXRD)

PXRD was conducted in Bragg–Brentano geometry on an Empyrean diffractometer (PANalytical) equipped with a curved Ge(111)‐monochromator using Cu‐K*α*_1_ radiation (*λ* = 1.5405 Å). For data analysis, the programs STOE WinXPow v.2.08^[^ ^55^ ^]^ and Jana2020^[^ ^53^ ^]^ were used.

##### Infrared (IR) Spectroscopy

IR spectroscopy was performed on a Bruker Vertex 70FTIR spectrometer with attenuated total reflection (ATR) accessory with 32 scans per measurement in a radiation range from 500 to 4000 cm^–1^. The program OPUS 6.5^[^ ^56^ ^,^ ^57^ ^]^ was used for data analysis.

##### NMR Spectroscopy

^1^H and ^13^C solid‐state NMR spectra were recorded with a Bruker Avance III 800 spectrometer. The ^13^C spectrum was acquired using a cross‐polarization pulse sequence at a MAS frequency of 7 kHz, while the ^1^H spectrum was collected at 10 kHz.

##### Quantum Chemical Calculations

DFT based calculations were performed using the Vienna Ab initio Simulation Package (VASP) v.6.4.2^[^ ^58^ ^,^ ^59^ ^]^ with the projector augmented wave (PAW) method and the Perdew–Burke–Ernzerhof (PBE) exchange‐correlation functional within the generalized gradient approximation (GGA).^[^ ^60^ ^]^ A plane‐wave cutoff energy of 500 eV was employed. The Brillouin zone was sampled using a Monkhorst–Pack^[^ ^61^ ^]^ *k*‐point mesh of 13  × 11 × 9, corresponding to a *k*‐point spacing of ≈0.010 Å^–1^. Input files for band structure calculations were created with VASPKIT.^[^ ^62^ ^]^ The structure was fully relaxed prior to all subsequent electronic structure calculations, with atomic positions and cell parameters optimized until the forces on each atom were below the convergence threshold.

Population analyses were carried out using the LOBSTER code (version 5.1.1),^[^ ^63^ ^]^ based on the wavefunction projections from the VASP calculations. The Bader analysis (version 1.05) was performed using the Bader code^[^ ^64^ ^]^ to evaluate charge distribution and bonding characteristics. For visualization, Vesta was used.^[^ ^65^ ^]^

The calculation of the IR spectrum was performed using the plotIR script.^[^ ^66^ ^]^

##### Toxicity Prediction

In silico toxicity prediction based on molecular similarity and machine‐learning methods was carried out with the online‐tool ProTox 3.0^[^ ^50^ ^]^ and the programs Toxtree^[^ ^49^ ^]^ and VEGA QSAR.^[^ ^51^ ^]^

## Conflict of Interest

The authors declare no conflict of interest.

## Supporting information

Supplementary Material

## Data Availability

The data that support the findings of this study are available from the corresponding author upon reasonable request.
