# Supplementary material for: Cyanoacetamidobetaine—A Zwitterionic Nitrile Derivative
Source: ChemistryOpen. 2025 Oct 9;15(4):e202500485. doi: 10.1002/open.202500485 (PMC13052206; doi:10.1002/open.202500485)
Supplement: Supplementary file 1 — Supplementary Material [file OPEN-15-e202500485-s001.pdf]

SUPPORTING INFORMATION

Cyanoacetamidobetaine - A Zwitterionic Nitrile Derivative

Pan Duan[1], Julia-Maria Hübner,[1*] Florian Puls[1], Vitaliy Romaka[1], Hans-Joachim Knölker[1,2], Michael Ruck[1,3]

[1] Faculty of Chemistry and Food Chemistry, TUD Dresden University of Technology, 01062 Dresden, Germany

[2] Sächsische Akademie der Wissenschaften zu Leipzig, Karl-Tauchnitz-Straße 1, 04107 Leipzig, Germany

[3] Max Planck Institute for Chemical Physics of Solids, Nöthnitzer Straße. 40, 01187 Dresden, Germany

1. Crystal Structure
2. Powder X-ray Diffraction (PXRD)
3. Infrared Spectroscopy (IR)
4. Nuclear magnetic resonance spectroscopy (NMR)
5. DFT Calculations

1. Crystal Structure

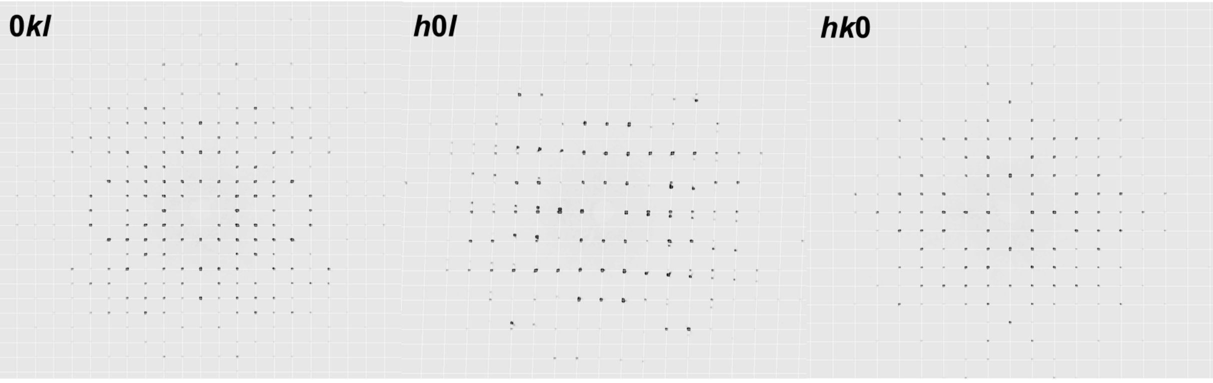

Figure S1. Reciprocal lattice reconstructions clearly showing twinning of the selected crystal.

Table S1. Data collection and crystallographic information for cyanoacetamidobetaine.

Composition	$(CH_3)_3N^+CH_2C(=O)N^-$–C≡N
Space group, Pearson symbol	$P2_1/c$ (No. 14), *mP*84
a / Å	7.3365(2)
b / Å	8.8351(3)
c / Å	11.0977(4)
β / °	91.948(2)
V / $Å^3$	718.92(4)
Formula units, *Z*	4
Density / (g cm^{-3})	1.30
Formula weight	141.2
Diffractometer	Rigaku Synergy S diffractometer with Mo-Kα radiation (λ = 0.71073 Å)
Measurement range	2.78 ≤ θ ≤ 30.18°<br>–9≤*h*≤10; –12≤*k*≤11; –15≤*l*≤15
Reflections collected / independent within $I>3\sigma(I)$ / restraints / parameters	2139/2072/0/92
Fourier difference ρ_{min}/ρ_{max} (electrons/$Å^3$)	–0.16/0.14
Residuals / GOF	R_1 = 0.039, wR_2 = 0.119 / 1.81

Table S2. Atomic coordinates and (equivalent) isotropic displacement parameters (in Å^2) for cyanoacetamidobetaine from single-crystal diffraction data refinement.

Atom	Site	*x*/*a*	*y*/*b*	*z*/*c*	U_{iso}/U_{eq}*
O1	4*e*	0.07642(7)	0.68252(6)	0.41359(5)	0.0231(2)
N1	4*e*	–0.14834(8)	0.86563(8)	0.42674(6)	0.0222(2)
N2	4*e*	0.26055(8)	0.85867(7)	0.23718(6)	0.0182(2)
N3	4*e*	–0.29468(9)	0.73988(8)	0.59979(6)	0.0228(2)
C1	4*e*	–0.2216(1)	0.79244(9)	0.51799(7)	0.0220(2)
C2	4*e*	0.4032(1)	0.86331(9)	0.33785(8)	0.0218(2)
C3	4*e*	0.2600(1)	0.70452(9)	0.17948(7)	0.0212(2)
C4	4*e*	0.00258(9)	0.80190(9)	0.38192(7)	0.0186(2)
C5	4*e*	0.07497(9)	0.90062(9)	0.28128(7)	0.0191(2)
C6	4*e*	0.3086(1)	0.97453(9)	0.14419(7)	0.0227(2)
H1C6	4*e*	0.3105	0.0733	0.1802	0.0273
H2C6	4*e*	0.4267	0.9520	0.1140	0.0273
H3C6	4*e*	0.2193	0.9722	0.0789	0.0273
H1C3	4*e*	0.2397	0.6288	0.2396	0.0254
H2C3	4*e*	0.1644	0.6996	0.1183	0.0254
H3C3	4*e*	0.3754	0.6869	0.1437	0.0254
H1C2	4*e*	0.3762	0.7879	0.3969	0.0261
H2C2	4*e*	0.5209	0.8435	0.3060	0.0261
H3C2	4*e*	0.4034	0.9615	0.3749	0.0261
H1C5	4*e*	–0.0117	0.9018	0.2149	0.0229
H2C5	4*e*	0.07565	0.00462	0.3064	0.0229

*Anisotropic displacement parameters U_{ani} are provided in Table S2.

Table S3. Coefficients U_{ij} of the tensor of the anisotropic displacement parameter (in Å^2) for cyanoacetamidobetaine.

Atom	U_{11}	U_{22}	U_{33}	U_{12}	U_{13}	U_{23}
O1	0.0222(3)	0.0216(3)	0.0255(3)	0.0026(2)	0.0029(2)	0.0023(2)
N1	0.0189(3)	0.0223(4)	0.0256(4)	0.0009(2)	0.0034(2)	–0.0005(3)
N2	0.0167(3)	0.0175(3)	0.0203(3)	0.0002(2)	0.0001(2)	–0.0002(2)
N3	0.0199(3)	0.0249(4)	0.0239(3)	0.0001(2)	0.0051(2)	–0.0019(3)
C1	0.0182(4)	0.0213(4)	0.0262(4)	0.0014(3)	–0.0010(3)	–0.0051(3)
C2	0.0179(4)	0.0234(4)	0.0237(4)	–0.0003(3)	–0.0032(3)	–0.0010(3)
C3	0.0227(4)	0.0177(4)	0.0233(4)	–0.0002(3)	0.0030(3)	–0.0021(3)
C4	0.0168(3)	0.0188(4)	0.0200(4)	–0.0017(3)	–0.0020(3)	–0.0023(3)
C5	0.0158(3)	0.0190(4)	0.0225(4)	0.0024(2)	0.0004(3)	0.0005(3)
C6	0.0229(4)	0.0195(4)	0.0260(4)	–0.0007(3)	0.0033(3)	0.0040(3)

Table S4. Selected interatomic distances and bond angles for cyanoacetamidobetaine.

Atoms	Distance / Å	Atoms	Bond angle / °
O1–C4	1.2314(9)	C2–N2–C3	109.41(5)
N1–C1	1.330(1)	C2–N2–C5	111.40(6)
C4	1.3519(9)	C2–N2–C6	108.51(5)
N2–C2	1.505(1)	C3–N2–C5	111.77(5)
C3	1.505(1)	C3–N2–C6	108.74(6)
C5	1.5086(9)	C5–N2–C6	106.89(6)
C6	1.504(1)	C1–N1–C4	115.76(7)
N3–C1	1.167(1)	N3–C1–N1	173.92(8)
C4–C5	1.527(1)	O1–C4–N1	127.56(7)
		O1–C4–C5	122.52(6)
		N1–C4–C5	109.93(6)
		N2–C5–C4	115.93(6)

2. Powder X-ray diffraction (PXRD)

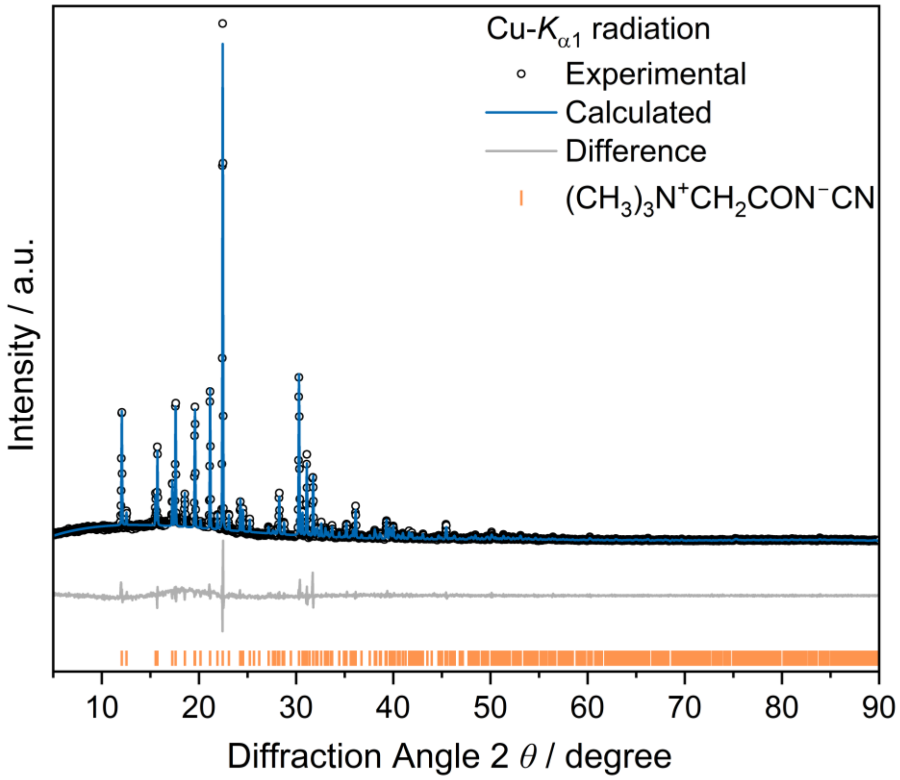

Figure S2. Powder diffractogram of cyanoacetamidobetaine obtained at room temperature with Cu-Kα_1 radiation.

3. Infrared Spectroscopy (IR)

Table S5. Assignment of IR bands of cyanoacetamidobetaine (Figure 5).

Wavenumber / cm^{-1}	Assignment	Remarks
3067, 3037, 3000 (weak)	=C–H or sp^2/sp^3 C–H stretches	represent CH_3 groups on the quaternary N (asymmetric stretch) and possibly the CH_2 linker
2951	Aliphatic C–H stretch	CH_3/CH_2 symmetric stretch
2133	Nitrile stretch (C≡N)	Strong and sharp, confirms presence of –C≡N group
1722	C=O stretch (carbonyl)	Corresponds to the ketone-like C=O in the acyl group. Shifted from ~1740 due to conjugation with the adjacent N^-
1606	N–C stretch or delocalized amide resonance	Possibly related to delocalized C–N/N^- interaction, common in amidinate-like systems.
1478, 1452	CH_3 bending (scissoring)	Symmetric and asymmetric bending of the three methyls on the N^+ group
1388	CH_3 umbrella bend or symmetric deformation	Often seen in quaternary ammonium compounds
1306, 1234	C–N stretches or in-plane CH wagging	Typical for C–N stretches, especially from quaternary ammonium and adjacent C–N bonds
1135	C–C or C–N stretch	Could be part of the CH_2–CO or CH_2–N^+ linkage
998, 913	CH_2 rocking or N–C–C wagging	Common for CH_2 adjacent to charged groups
779, 754	CH out-of-plane bending	Possibly from CH_2 or CH_3 deformations
677, 625	C–N deformation or lattice-like bending	Often seen in quaternary ammonium salts and substituted nitriles

4. Nuclear magnetic resonance spectroscopy (NMR)

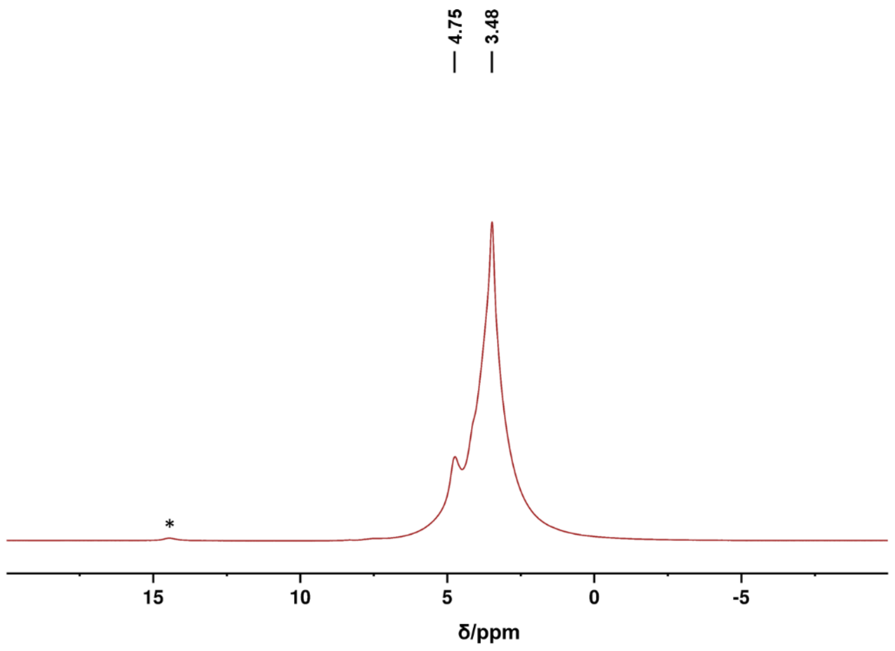

Figure S3. ^{1}H MAS NMR spectrum of cyanoacetamidobetaine. Note: *spinning side bands.

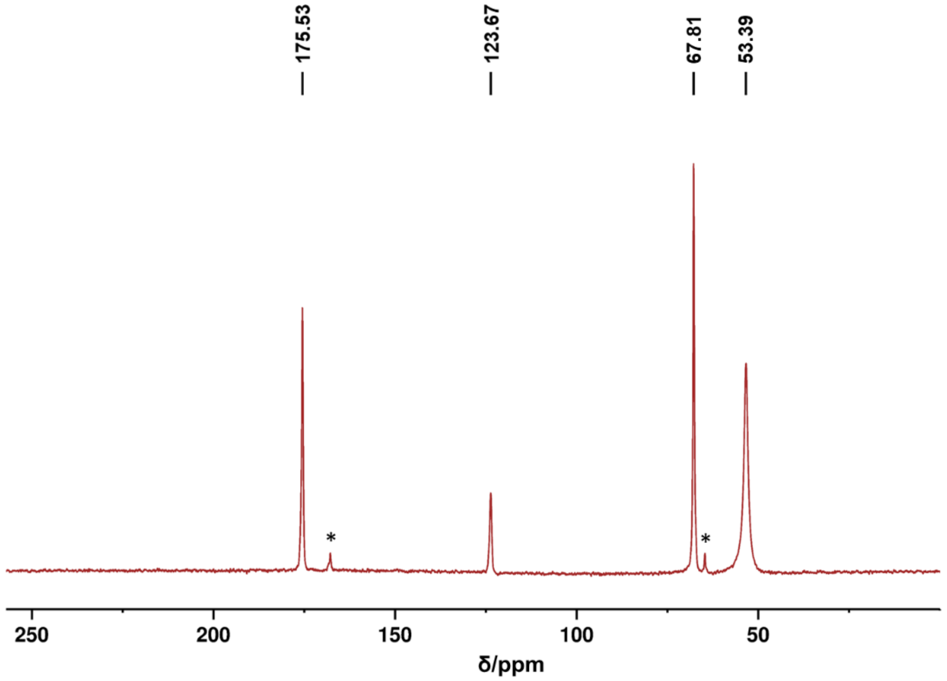

Figure S4. ^{13}C CP/MAS NMR spectrum spectra of cyanoacetamidobetaine. Note: *spinning side bands.

Solid-state ^{1}H MAS NMR (800 MHz, 10 kHz MAS, ppm): δ = 3.48 (s, 9H, CH_3), 4.75 (s, 2H, CH_2); Solid-state ^{13}C CP/MAS NMR (200 MHz, 7 kHz MAS, ppm): δ = 53.39 (CH_3), 67.81 (CH_2), 123.67 (C≡N), 175.53 (C=O).

5. DFT Calculations

Table S6. Crystal structure of cyanoacetamidobetaine after full geometry optimization (a = 7.4624 Å, b = 9.07376 Å, c = 11.33964 Å, β = 91.1689°).

Atom	Site	*x*/a	*y*/b	*z*/c
O1	4*e*	0.0828	0.6835	0.4138
N1	4*e*	0.8559	0.8595	0.4277
N2	4*e*	0.2594	0.8581	0.2377
N3	4*e*	0.6996	0.7487	0.5980
C1	4*e*	0.7792	0.7949	0.5163
C2	4*e*	0.3995	0.8635	0.3351
C3	4*e*	0.2603	0.7080	0.1809
C4	4*e*	0.0063	0.8000	0.3825
C5	4*e*	0.0763	0.8968	0.2827
C6	4*e*	0.3069	0.9712	0.1461
H1C6	4*e*	0.3083	0.0798	0.1884
H2C6	4*e*	0.4400	0.9428	0.1137
H3C6	4*e*	0.2064	0.9676	0.0744
H1C3	4*e*	0.2331	0.6259	0.2486
H2C3	4*e*	0.1570	0.7065	0.1111
H3C3	4*e*	0.3930	0.6923	0.1432
H1C2	4*e*	0.3693	0.7776	0.3992
H2C2	4*e*	0.5305	0.8451	0.2957
H3C2	4*e*	0.3946	0.9728	0.3759
H1C5	4*e*	0.9817	0.8922	0.2070
H2C5	4*e*	0.0838	0.0114	0.3132

Table S7. Net charges on individual atoms as determined by Bader charge analysis of the DFT-relaxed structure of cyanoacetamidobetaine.

Atom	Environment	Net charge
O1	Double bonded to C	–1.17
N1	Between two C	−1.26
N2	Quarternary N tetrahedrally surrounded by sp^3-hybridized C	+0.92
N3	Terminal N	−1.28
C1	Between two N	+1.44
C2	sp^3-hybridized C	+0.14
C3	sp^3-hybridized C	+0.11
C4	Double bonded to O	+1.41
C5	sp^3-hybridized C	+0.18
C6	sp^3-hybridized C	+0.11

Table S8. Calculated interatomic distances, and ICOBI, and –ICOPH values obtained from LOBSTER analysis for the DFT-relaxed structure of cyanoacetamidobetaine.

**Atoms**	**Distance / Å**	**ICOBI at E_F**	**-ICOPH at E_F / eV**
O1	– C4: 1.24899	1.57	16.29
N1	– C1: 1.30552	1.36	14.18
	– C4: 1.35523	1.26	12.30
N2	– C5: 1.50984	0.93	9.06
	– C6: 1.50730	0.94	8.91
	– C2: 1.50572	0.94	8.92
	– C3: 1.50681	0.93	8.92
N3	– C1: 1.18705	2.51	19.72
C1	– N1: 1.30552	1.36	14.18
	– N3: 1.18705	2.51	19.72
C2	– N2: 1.50572	0.94	8.92
C3	– N2: 1.50681	0.93	8.92
C4	– N1: 1.35523	1.26	12.30
	– C5: 1.53312	0.95	9.44
	– O1: 1.24899	1.57	16.29
C5	– N2: 1.50984	0.93	9.06
	– C4: 1.53312	0.95	9.44
C6	– N2: 1.50730	0.94	8.91

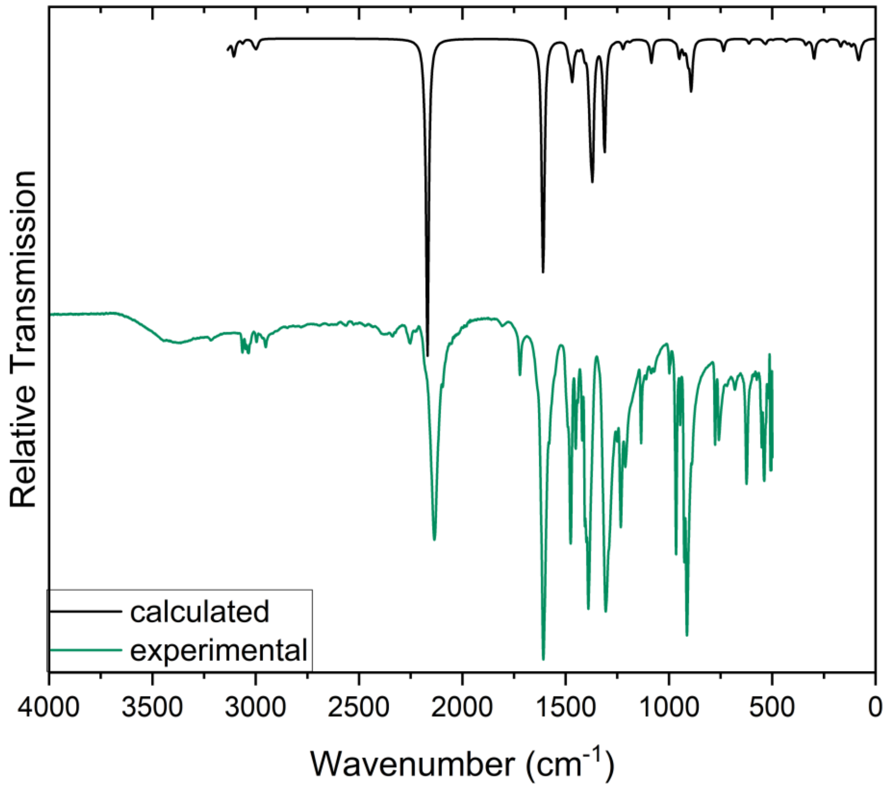

Figure S5. DFT calculated and experimental IR spectra of cyanoacetamidobetaine.
